# Complex Systems and Fractional Dynamics

**DOI:** 10.3390/e20070507

**Published:** 2018-07-03

**Authors:** António M. Lopes, J. A. Tenreiro Machado

**Affiliations:** 1 UISPA–LAETA/INEGI, Faculty of Engineering, University of Porto, R. Dr. Roberto Frias, 4200-465 Porto, Portugal; 2 Institute of Engineering, Polytechnic of Porto, Department of Electrical Engineering, R. Dr. António Bernardino de Almeida, 431, 4249-015 Porto, Portugal

**Keywords:** dynamics, complex systems, fractional calculus, entropy, information

Complex systems (CS) are pervasive in many areas of science and technology, namely in financial markets, transportation, telecommunication and social networks, world and country economies, immunological systems, living organisms, computational systems, and electrical and mechanical structures. CS are often composed of a large number of interconnected and interacting entities, exhibiting a much richer global scale dynamics than their individual parts.

This Special Issue focuses on original and new research results on CS and fractional dynamics. It comprises 12 selected manuscripts that address novel issues, as well as specific topics illustrating the broad impact of entropy and information theory-based techniques in complexity, nonlinearity, and fractionality. In the following, the manuscripts are presented in alphabetical order.

In the paper “Analytical Approximate Solutions of (n+1)-Dimensional Fractal Heat-Like and Wave-Like Equations”, Omer Acan, Dumitru Baleanu, Maysaa Mohamed Al Qurashi, and Mehmet Giyas Sakar propose a new type of (n+1)-dimensional reduced differential transform method (RDTM) based on a local fractional derivative (LFD) to solve (n+1)-dimensional local fractional partial differential equations (PDEs) in Cantor sets. Firstly, the authors introduce the theoretical concepts. Afterwards, they apply the method to the (n+1)-dimensional fractal heat-like equations (HLEs) and wave-like equations (WLEs). The authors conclude that the new technique is efficient and easy to apply [1].

The paper “A Novel Numerical Approach for a Nonlinear Fractional Dynamical Model of Interpersonal and Romantic Relationships”, by Jagdev Singh, Devendra Kumar, Maysaa Al Qurashi, and Dumitru Baleanu, proposes the *q*-homotopy analysis Sumudu transform method (*q*-HASTM) to obtain the approximate solution for the nonlinear fractional dynamical model of interpersonal and romantic relationships. The results obtained by employing the proposed scheme reveal good accuracy, effectiveness, flexibility, and simplicity [2].

In the paper “Fractional Derivative Phenomenology of Percolative Phonon-Assisted Hopping in Two-Dimensional Disordered Systems”, by Renat Sibatov, Vadim Shulezhko, and Vyacheslav Svetukhin, the anomalous advection-diffusion in two-dimensional semiconductor systems with coexisting energetic and structural disorder is described in the framework of a generalized model of multiple trapping on a comb-like structure. To validate the model, the authors compare the analytical solutions with the results of a Monte Carlo simulation of phonon-assisted tunneling in two-dimensional patterns of a porous nanoparticle agglomerate and a phase-separated bulk heterojunction. The variations of the anomalous advection-diffusion parameters as functions of the electric field intensity as well as levels of energetic and structural disorder are also presented [3].

In “Fractional Jensen–Shannon Analysis of the Scientific Output of Researchers in Fractional Calculus”, J. A. Tenreiro Machado and António M. Lopes analyze the citation profiles of researchers in fractional calculus. Different metrics are used to quantify the dissimilarities between the data, namely the Canberra distance and the classical and the generalized (fractional) Jensen–Shannon divergence. The information is visualized by means of multidimensional scaling and hierarchical clustering. The mathematical tools and metrics allow for direct comparison and visualization of researchers based on their relative positioning and on patterns displayed in 2- or 3-dimensional maps [4].

In “Fractional-Order Identification and Control of Heating Processes with Non-Continuous Materials”, Riccardo Caponetto, Francesca Sapuppo, Vincenzo Tomasello, Guido Maione, and Paolo Lino present a fractional order model of a heating process and a comparison of fractional and standard PI controllers in its closed loop system. The modeling results confirm the fractional nature of the heating processes when diffusion occurs in non-continuous composite materials, and show how the fractional order can be used as a characteristic parameter for non-continuous materials with different composition and structure. Moreover, the authors compare three different kinds of controllers in order to keep the beam temperature constant at a fixed length [5].

The manuscript “Information Technology Project Portfolio Implementation Process Optimization Based on Complex Network Theory and Entropy”, by Qin Wang, Guangping Zeng, and Xuyan Tu, introduces an optimization method based on complex network theory and entropy, for assisting portfolio managers in recognizing the structure of the portfolio and determining the cooperation range. Firstly, a complex network model for an information technology project portfolio is constructed, where the project is simulated as an artificial life agent. Then, social network analysis is used to detect and divide communities in order to estimate the roles of projects between different portfolios. Based on these, the efficiency and the risk are measured using entropy and are balanced by searching for adequate hierarchy community divisions [6].

In the work “Minimum Sample Size for Reliable Causal Inference Using Transfer Entropy”, Antônio M. T. Ramos and Elbert E. N. Macau investigate the concept of transfer entropy to determine the minimum sample size that produces a reliable causal inference between variables. The methodology is applied to two prototypical models: the linear autoregressive-moving average and the non-linear logistic map. The relationship between the transfer entropy value and the sample size is systematically examined. The methodology is shown to offer a realistic lower bound for the sample size to produce a reliable outcome [7].

In “Multiplicity of Homoclinic Solutions for Fractional Hamiltonian Systems with Subquadratic Potential”, by Neamat Nyamoradi, Ahmed Alsaedi, Bashir Ahmad, and Yong Zhou, the authors study the existence of homoclinic solutions for fractional Hamiltonian systems with left and right Liouville–Weyl derivatives. They establish some new results concerning the existence and multiplicity of homoclinic solutions for the given system by using Clark’s theorem from critical point theory and fountain theorem [8].

In the paper “On the Modelling and Control of a Laboratory Prototype of a Hydraulic Canal Based on a TITO Fractional-order Model”, by Andres San-Millan, Daniel Feliu-Talegón, Vicente Feliu-Batlle, and Raul Rivas-Perez, the authors develop a two-input, two-output (TITO) fractional order mathematical model of a laboratory prototype of a hydraulic canal. This canal is made up of two pools that have a strong interaction between them. The inputs of the TITO model are the pump flow and the opening of an intermediate gate, and the two outputs are the water levels in the two pools. Experiments show that the proposed model is accurate in capturing the canal dynamics [9].

The article “Research Entropy Complexity About the Nonlinear Dynamic Delay Game Model”, by Xueli Zhan, Junhai Ma, and Wenbo Ren, proposes a double oligopoly market model of renewable energy, and analyses the complex dynamic characteristics of the system based on entropy and chaos theory. The results show that with the increase of the natural growth rate of energy, the stability of the system is not affected, but the market demand of oligopoly 1 is gradually reducing and the market demand of the oligopoly 2 is gradually increasing [10].

In “Toward a Theory of Industrial Supply Networks: A Multi-Level Perspective via Network Analysis”, Yi Zuo and Yuya Kajikawa reconceptualize supply chains (SCs) as supply networks (SNs) in complex adaptive systems (CASs). First, the authors propose an integrated framework of a CAS network by synthesizing multi-level network analysis from the network-, community-, and vertex-perspectives. Second, in order to emphasize the CAS properties of SNs, they conduct a real-world SN based on the Japanese industry and describe an advanced investigation of SN theory. Third, the authors formulate a quantitative metric of entropy to measure the complexity and robustness of SNs. The results not only support a specific understanding of the structural outcomes relevant to SNs, but also deliver efficient and effective support to the management and design of SNs [11].

The paper “Two Approaches to Obtaining the Space-Time Fractional Advection-Diffusion Equation”, by Yuriy Povstenko and Tamara Kyrylych, discusses two approaches that result in two different generalizations of the space-time-fractional advection-diffusion equation. The Caputo time-fractional derivative and Riesz fractional Laplacian are used. The fundamental solutions to the corresponding Cauchy and source problems in the case of one spatial variable are studied using the Laplace transform with respect to time, and the Fourier transform with respect to the spatial coordinate. Several numerical results are illustrated graphically [12].

In conclusion, the guest editors hope that the selected papers will help scholars and researchers to push forward the progress in the emerging area of Complex Systems and Fractional Dynamics.
